# Current Evidence for the Use of Smoflipid® Emulsion in Critical Care Patients for Parenteral Nutrition

**DOI:** 10.1155/2018/6301293

**Published:** 2018-11-21

**Authors:** Alberto A. Leguina-Ruzzi, Rina Ortiz

**Affiliations:** ^1^Institute of Physiology AS CR, v.v.l., Department of Mitochondrial Physiology, Vídeňská 1083, 14220 Prague 4, Czech Republic; ^2^Centro de Biotecnología, Universidad Técnica Federico Santa María, Avenida España, 1680 Valparaíso, Chile; ^3^Universidad Bernardo O Higgins, Facultad de Salud, Departamento de Ciencias Químicas y Biológicas, Santiago, Chile

## Abstract

There are strong data showing that malnutrition is highly prevalent in intensive care unit patients (20–50% in the worldwide), presenting a negative accumulated body energy balance. This results in an increased mortality, infections, and hospital length stay with high costs associated with the total treatment. Parenteral nutrition is the first option when the patient's physical condition is not suitable for oral nutrient intake. It is composed essentially by lipids as an energy source, metabolic, and structural function. However, these patients also require a mixture of essential and nonessential fatty acids (SMOF emulsions) to supply not only energy needs but also restore immunological, anti-inflammatory, and proregenerative functions. A revision of the safety and efficacy of Smoflipid® in patients requiring long-term parenteral nutrition was discussed here. Although controversial data are available indicating the contraindications or effectiveness of its use, most of studies presented indicate favorable benefits associated with improved clinical outcomes. The reported roles of this supplementation include positive immunomodulatory and anti-inflammatory effects, positive impact in liver function, reduction of hospital stay, and nosocomial infections as additional contributions to its energetic role, which in many cases results in reduced total costs per patient. Finally, many authors propose that the use of Smoflipid® should become a gold standard of parenteral nutrition in intensive unit care patients and that the costs associated with this supplement should not be limiting for its use, not only to improve the clinical outcome but also to reduce the treatment costs.

## 1. Introduction

There is alarming evidence showing that malnutrition is highly prevalent in ICU patients [1]. The diagnosis of malnutrition has been challenging due to the absence of a unifying definition; however, it is accepted that malnutrition is associated with a negative accumulated energy balance [2]. This imbalance significantly increases mortality, nosocomial infections, and hospital LOS [3], generating an increase in the costs associated with the treatment [4]. In recent years, it has been reported that the worldwide prevalence of this condition is between 20 and 50% [5].

PN is the most common alternative intervention when the patient's physical conditions are not suitable for oral (EN) nutrient intake [6]. Its composition and lipids are indispensable as an energy source, metabolic, and structural mediator [7]. It is important to highlight that these patients require a mixture of essential and nonessential fatty acids, currently called SMOF emulsions, to supply not only for energy needs but also immunological, anti-inflammatory, and proregenerative capacities [8].

The oil sources of this type of emulsion are coconut (30%), soybean (30%), olive (25%), and fish (15%), being the most complete supplement currently in the market as compared in Table 1 [9, 10].

Emulsions enriched in PUFA n-3 fatty acids are recommended by the international guidelines for the management of critical patient [11]. Previous studies have shown that supplementation of these fatty acids is safe and confers an immunomodulatory and anti-inflammatory effect as further contribution to its energetic role [12]. In addition, other potential therapeutic applications of lipid emulsions are cell structural function and proliferation, provide sufficient fatty acids, improve metabolism and limit/reverse energy deficit, modulate oxidative stress, limit the contribution of lipid peroxidation to oxidative stress, maintain or increase antioxidant concentrations and intrinsic immune function, support the immune system and limit immunosuppression, reduce the incidence of infectious complications, resolve inflammation, and prevent/regulate hyperinflammation, especially important for patients with preexisting inflammation (e.g., surgery, sepsis, and chronic inflammatory diseases) [13].

The main contraindications to its use are hypersensitivity to fish, egg, soybean, or peanut protein or to any of the active ingredients or excipients (infrequent), severe hyperlipidemia (frequent but transitory in ICU patients), severe liver insufficiency (frequent), severe blood coagulation disorders (highly controlled in ICU), and severe renal insufficiency (frequent) without access to hemofiltration or dialysis [7]. For this reason, a continuous and rigorous monitoring is pivotal for the proper nutritional supplementation.

Despite the proven beneficial effects of lipid emulsions enriched in PUFA n-3, MCT, and LCT, there is lack of knowledge about its cost-effectiveness associated with the improvement of the most relevant clinical outcomes. Two recent studies evaluated the economic drug aspect of Smoflipid® (Fresenius Kabi) compared to PN nutrition parenteral without this lipid emulsion.

### 1.1. Molecular Basis for the Use of Lipids in Nutrition

Dietary fat in humans mainly consists of triacylglycerols, which are molecules having three fatty acids esterified to glycerol. Phospholipids, glycolipids, and cholesterol are quantitatively less important in the diet but play vital functions in the body [14].

The fatty acids are classified as saturated, monounsaturated, and polyunsaturated. Whereas saturated and monounsaturated can be synthesized in the body, two simpler polyunsaturated fatty acids (PUFAs), linoleic acids and *α*-linolenic acids, can be produced in plants, bacteria, and fungi. They are important to human life and need to be part of the diet (essential fatty acids). The human body is able to convert them into other PUFAs with longer chain lengths and more double bonds [15].

Most of the lipid mass in the body, presented as triacylglycerols in the adipose tissue, acts as an important reserve of available metabolic energy (storage fat). It also contains small quantities of cholesteryl esters and fat-soluble vitamins. The fatty acid composition of triacylglycerols in the adipose issue is related to the composition of the fatty acid in the diet [16].

The nature of phospholipid fatty acids determines the properties of the biological membranes as the fluidity of the lipid bilayer. However, this function also depends on the ratio of phospholipids and cholesterol and the interactions between phospholipids and membrane proteins as transporters, receptors, antigens, or nutrients. Because the membrane fluidity is critical for these functions, it is regulated by slight changes in the proportion of phospholipids, cholesterol, and fatty acids. For this reason, the composition of structural lipids in membranes is conservative in contrast to that of storage, which can present a wide range of possibilities. Both structural and storage pools of lipids are continually being replaced as fat stores and used as body fuels. However, structural lipids are transformed into a variety of metabolic end products, mainly eicosanoids, which act as regulators of cell metabolism [14].

The energy supply of triacylglycerols in the body is approx. 38 kJ/g, higher than proteins (approx. 17 kJ/g) and carbohydrates (approx. 16 kJ/g). As storage fuel, triacylglycerols can be stored in anhydrous form having more energy for less bulk than complex highly hydrated polysaccharides (food starches and body glycogen) [14].

The useful and metabolizable energy available mainly depends on the chain length of the fatty acids. Saturated (and to a lesser extent unsaturated) fatty acids with chain lengths above eighteen carbons are less digested and absorbed, with efficiency decreasing as chain length increases. The energy values of medium- and short-chain fatty acids are considerably lower than those of the longer chain, and this phenomenon is advantageous in the manufacture of reduced-calorie foods [14].

Lipids not only serve as building blocks for the cell membrane and fuel source but also function as mediators with the ability to influence the immunity. Proinflammatory lipid mediators as prostaglandins and leukotrienes are generated from arachidonic acid (AA), a key member of the n-6 PUFAs (present in the soybean oil) [17]. In contrast, lipid mediators derived from the n-3 fatty acids, eicosapentaenoic acid (EPA), or docosahexaenoic acid (DHA) present in fish oil may exhibit anti-inflammatory properties [14, 17]. Studies have proposed that modulating the amount of PUFA, the n-6/n-3 ratio and the composition of lipid emulsions may be useful to improve the outcome of critically ill patients [18].

It is important to highlight that it has been described that during critical conditions, patients experience a dramatic depletion of PUFA *n*-3/*n*-6. This abnormality leads to a “conditional” essential fatty acid deficiency that directs supplementation with PUFA that could correct in critical care patients [19].

### 1.2. Current Clinical Evidence in the Use of SMOF Emulsions

One of the first studies from Grimm et al. showed in postoperative double-blind randomized setting that supplementation of Smoflipid® is well-tolerated, modulating FA, and leukotriene patterns, suggesting favourable anti-inflammatory effects and further clinical benefits. Furthermore, it reduces the LOS in around 35% without altering laboratory measurements [20].

In the same year, Mertes et al. [21] tested in a prospective, double-blind European multicenter study the safety, tolerance, metabolic, and clinical efficacy in postsurgical patients. Results showed that the supplementation of Smoflipid® 1.5 g/kg/day does not affect triacylglycerols phospholipids and total cholesterol levels. Parameters such as hematological, clinical biochemistry, coagulation profile, and clinical course (arterial blood pressure, heart rate, and body temperature) showed no change in response to this lipid emulsion. The authors concluded that the use of Smoflipid® is associated with a better liver tolerance and a shorter length of hospitalization.

In discrepancy with the previous findings, in 2012, it was reported that the use of Smoflipid® versus soybean emulsion did not confer any benefit in terms of inflammatory markers, neither improves clinical outcomes. More contradictory, this report shows no changes in LOS and an increase on postoperative complications such as anastomosis leaks, postoperative ileus, and abdominal abscess [22].

A year later, Klek et al. demonstrated long-term (4 weeks) safety. The authors showed that patients under Smoflipid® supplementation with PN presented lower levels of alanine transaminase, aspartate transaminase, and total bilirubin, in addition to less than grade “4” (serious) adverse events and an increase in eicosapentaenoic acid, docosahexaenoic acid, *n*-3/*n*-6 fatty acid ratio, and serum *α*-tocopherol concentrations. However, no changes were observed on IL-6 and sTNF-RII levels [23].

A small prospective, randomized, and double-blinded study in an Egyptian postopera,tive ICU showed that Smoflipid® reduces the levels of IL-6 after one week of supplementation. However, no changes were observed in clinic outcomes such as length of hospitalization, duration of stay in ICU, duration of mechanical ventilation, or mortality. In addition, its use did not affect lipids profile or vital signs [24]. In concordance, a study of a Taiwanese ICU population showed that Smoflipid® had a better triglyceride-lowering effect compared to MCT/LCT in adult patients undergoing gastrointestinal surgery. However, no changes were observed in proinflammatory markers (CRP, IL-6, IL-10, TNF-*α*, and TGF-*β*1) and oxidative stress (ROS and superoxide) [25].

In 2017, five key publications gave important evidence about the use of Smoflipid® in ICU patients.

Mundi et al. explore the benefits and risks of different fatty acid sources from 13 prospective clinical trials with Smoflipid® in adult and pediatric patients. The authors highlight that emulsion is more physiologically similar to normal dietary human consumption and human milk than isolated lipid sources. The multiple sources help to give a balanced nutritional input, increasing the advantages and reducing the disadvantages. The main conclusions of the study are that Smoflipid® has a positive impact on liver enzymes due low phytosterol and high vitamin E content, in addition to decrease in lipid peroxidation and improvement on *ω*-3 to *ω*-6 PUFA ratio, producing a less proinflammatory profile [26].

Another case report in ICU septic patients requiring PN supplemented the nutrition with Smoflipid® 104.6 kJ/kg/d, 2 g amino acids/kg/d, and 0.8 g/lipids/kg/d. This setting was maintained for 24 days starting on the second day after operation. They evidenced that postoperative PN with SMOFlipid enabled the provision of adequate energy, and Smoflipid was well tolerated in this patient during a period of sepsis [27].

Hurt et al. proved that Smoflipid® is well tolerated during long-term administration (246 days, 7 days a week), having a positive impact on liver function. Moreover, the biopsies showed proven IFALD with elevated total bilirubin (41.04 *µ*mol/L) after 11 years of long-term home PN with Intralipid®. In the interventional setting, after change from Intralipid® to Smoflipid®, the authors observed weight gain (5 kg in/10 months), decrement in total bilirubin after 1 month (from 41.04 *µ*mol/L to 13.6 *µ*mol/L), normalized transaminases (AST, 48 U/L; ALT, 40 U/L), no more abdominal pain or discomfort in the abdominal area, and no further episodes of acute pancreatitis [28].

Another case report assessed long-term effects of low-dose Smoflipid® (100 g/week) on liver function in patients with PN dependency due to extreme SBS and high output fistula, scheduled for combined intestinal and liver transplant. After 7 months, progressive IFALD developed, with extremely high total bilirubin (172.71 *µ*mol/L). After lipid emulsion change, it was evident that a sustained gradual decrease in serum total bilirubin 4 weeks after initiating Smoflipid®, and later the liver biopsy at week 20 on Smoflipid® revealed markedly improved cholestasis. At week 23, the patient's total bilirubin nearly normalized to 0.9 mg/dL, the jaundice and hepatomegaly were resolved, patient's listing could be changed to an isolated intestine transplant, and at the time of transplant, serum total bilirubin was normalized (13.6 *µ*mol/L) and liver enzymes were decreased. The authors suggest that Smoflipid® may have a role as therapy to improve liver biochemistry even in advanced IFALD. However, it is essential to note that improvement in IFALD occurred while the patient was maintained on a restricted dose of Smoflipid® [29].

Another case report from 2017 in patients under need of TPN due to PSC and UC evaluated the effects of replacing Intralipid® with Smoflipid® on liver enzymes, specifically GGT. Results obtained showed weight stabilization enabling TPN reduction to 3-4 days per week, with fewer carbohydrates and including Smoflipid® in every TPN bag. Within two months, GGT levels, a potential marker of oxidative stress, completely normalized, and patient regained the ability to participate in family activities, work full-time, and take care of his house and yard [30].

Recently, a comparative study in preterm infants (mean gestational age 26.7 weeks) requiring PN for >14 days evaluated as primary outcome mortality and rates of severe neonatal morbidities. The authors reported lower incidence of late onset sepsis and greater weight at 36 weeks before conception with Smoflipid® versus Intralipid, in addition to less retinopathy of prematurity (ROP) and greater rates of intraventricular hemorrhage (any grade) with Intralipid.

No significant difference in mortality or rates of any other severe neonatal morbidity was documented. This study concludes that Smoflipid® was well tolerated in preterm infants and proved to be beneficial in terms of growth and incidence of sepsis. Moreover, SMOFlipid is fast becoming the primary lipid emulsion of choice replacing soybean-based formulations in neonatal units around the world [31].

Finally, a systematic review and meta-analysis on the effects of parenteral supplementation with *ω*-3 fatty acids on immune function and clinical outcomes in postoperative GI cancer patients that included 7 trials (457 participants) concluded that the use of SMOFlipid reduced incidence of infectious, shortened length of hospital stay, and increased level of CD4+ cells. Data suggested that parenteral *ω*-3 fatty acid supplementation is effective in improving the immune function and clinical outcomes of GI-cancer patients [32].

### 1.3. New Emerging Pharmacoeconomic Evidence

First, an observational and predictive study in China concluded that Smoflipid® significantly reduces nosocomial infections, generating a reduction in the days of hospitalization. Moreover, total costs per patient are reduced by 30%, considering the higher price for this supplement lipid. It should be noted that the effectiveness of Smoflipid® was observed in over 96% of the patients studied and generated a 80% cost reduction at hospital level. The subsequent evaluation elucidated that observed changes must be mainly due to reduction in the use of antibiotics and the costs of managing infections [33].

Second, a multicenter study corroborated these observations obtained and reported that the use of Smoflipid® prevents nosocomial infections by 35.6%, also the hospital ICU length stay was reduced, in particular in ICU, by over 10%. Furthermore, it was evidenced that PN parenteral nutrition with this lipid emulsion increases in 83.7% the probability of transfer from ICU to the general ward, and a 95% increased probability of early discharge. Additionally, the probability of death in the ICU reduces to 16.3% and of mortality in ward to 5%.

The analysis of global hospital costs showed that they are reduced by 20% mainly associated with the lower use of antibiotics and antibacterial interventions. The parameter ICER shows a strong dominance towards the use of emulsion enriched in omega-3 [34].

## 2. Conclusions

Research during the last decade has provided important knowledge in the use of third-generation lipid emulsions. Smoflipid® has shown an important advance for the nutrition in patients needing PN.

The pharmacoeconomic evaluation of the emulsion enriched in PUFA n-3 in parenteral nutrition in different Chinese ICUs indicates that it significantly improves clinical outcomes, along with a reduction in treatment costs. These observations are mainly due to the reduction of hospital stay and nosocomial infections. This would benefit 93% of the ICU patients, creating a profit scenario for health providers and for patients, inducing a recovery pattern accelerated and a subsequent early discharge.

Other nutritional supplements such as glutamine have been suggested as beneficial for critical care pathophysiological condition [35] and also in the economical aspect [36]; however, for the use of SMOF emulsions, no controversial evidence has emerged that could limit its use. Moreover, the recent approval by the FDA for its use in the United States will widely benefit the ICU units over the continent.

Reports reviewed here consider that the use of Smoflipid® should become a gold standard of parenteral nutrition. It has been suggested that should the use of this emulsion become a routine, costs associated when administered to patients where it is prescribed will generate not only economic utilities but also clinical convenience.

## Figures and Tables

**Table 1:** Comparison between intravenous fat emulsions commercially available (10 g fat/100 mL) (modified from [10]).

	Intralipid®	ClinOleic®	Smoflipid®	Omegaven®
Soybean (g)	10	2	3	0
MCT (g)	0	0	3	0
Olive oil (g)	0	8	2.5	0
Fish oil (g)	0	0	1.5	10
Alpha-tocopherol (mg/L)	38	32	200	150–296
Phytosterols (mg/L)	348 ± 33	327 ± 8	47.6	0

## Abbreviations

ALT: Alanine transaminase
AST: Aspartate transaminase
EN: Enteral nutrition
FA: Fatty acids
FDA: Food and drug administration
GGT: Glutamyl transpeptidase
GI: Gastrointestinal
ICER: Incremental cost-effective ratio
ICU: Intensive care unit
IFALD: Intestinal failure-associated liver disease
LCT: Long-chain triglyceride
LOS: Length of stay
MCT: Medium-chain triglyceride
PN: Parenteral nutrition
PSC: Primary sclerosing cholangitis
PUFA n-3: n-3 Polyunsaturated fatty acids
SBS: State behavioural scale
UC: Ulcerative colitis.
